# Quick and sustained clinical response to MEK inhibitor I in a NF1 patient with neurofibromas

**DOI:** 10.3332/ecancer.2018.862

**Published:** 2018-08-28

**Authors:** Honoré Papalia, Frédérique Audic, Gabriel Revon Rivière, Arnauld Verschuur, Nicolas André

**Affiliations:** 1 Department of Paediatric Haematology and Oncology, AP-HM, La Timone Children’s Hospital, Marseille 13005, France; 2 Department of Paediatric Neurology, AP-HM, La Timone Children’s Hospital, Marseille 13005, France; 3 Centre d’Essais Précoce en Cancérologie de Marseille, AP-HM, La Timone Children’s Hospital, Marseille 13005, France; 4 Aix-Marseille Univ, Institut Paoli-Calmettes, SMARTc, Marseille 13273, France

**Keywords:** MEK, children, neurofibromatosis, neurofibroma, glioma

## Abstract

Neurofibromatosis 1 (NF1) is an autosomal dominant tumour predisposition disorder with a birth incidence of about 1 in 2,700 and prevalence of 1 in 4,560. The NF1 gene codes for an ubiquitous protein: neurofibromin. Neurofibromin interacts with the proto-oncogene RAS to suppress tumour formation. Individuals with germline inactivation of the *NF1* gene have a propensity to develop both benign and malignant tumours.

We report the case of a 12-year-old child with NF1, diagnosed at the age of 15 months, for whom the clinical course has been marked by the appearance of multiple cutaneous and paraspinal neurofibromas responsible for impaired walking, motor deficiency and pain. A treatment with an MEK inhibitor, trametinib, was initiated and led to a quick and sustained clinical response.

## Introduction

Neurofibromatosis 1 (NF1) is an autosomal dominant tumour predisposition disorder that affects about 1 in 3,000 people [1]. The NF1 gene is located on chromosome 17q11.2. It encodes for neurofibromin, a cytoplasmic protein that is expressed in the nervous system [2]. Neurofibromin negatively regulates proto-oncogene RAS and acts as a tumour suppressor through the reduction of cell proliferation and differentiation by preventing the activation of downstream signalling pathways such as PI3K/AKT/mTOR and Raf/Mapk/MEK/ERK [3]. As a result, an individual with NF1 has a predisposition to develop both benign and malignant tumours. The characteristic lesion is benign neurofibroma, but there is also an 8%–13% lifetime risk of developing malignant peripheral nerve sheath tumour [2].

Surgical removal of neurofibromas is not always feasible due to tumour location, resulting in significant morbidity. In a clinical phase I study of selumetinib in NF1-associated inoperable plexiform neurofibromas, promising activity has been observed [4].

We report a case of a patient with NF1-associated neurofibromas who experienced substantial and sustained clinical benefit from the MEK inhibitor trametinib.

## Case report

We report the case of a 12-year-old child with NF1. This diagnosis was made at the age of 15 months based on clinical and imaging criteria according to the National Institutes of Health’s Consensus Development Conference Statement. There were 12 café-au-lait spots larger than 0.5 cm on the whole body. Initial cerebral magnetic resonance imaging (MRI) described an enlargement of the intracranial segments of both optical nerves compatible with glioma of the optical pathways, but with good and preserved visual function.

**Figure 1.**
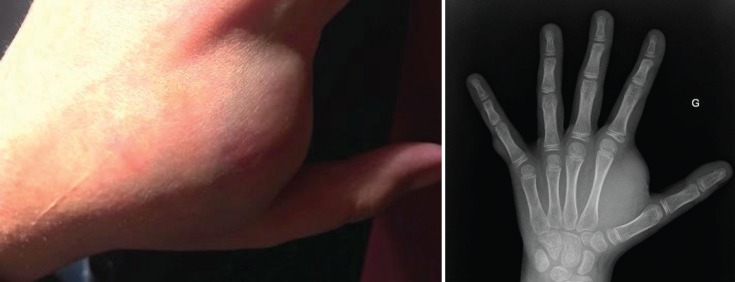
Subcutaneous neurofibroma (left hand): photography and X-ray.

The clinical course has been marked by the appearance of multiple asymptomatic cutaneous neurofibromas and one large subcutaneous neurofibroma at the first dorsal interosseous space of the left hand measuring 3 cm of the long axis, leading to difficulty in using this hand (Figure 1).

**Figure 2.**
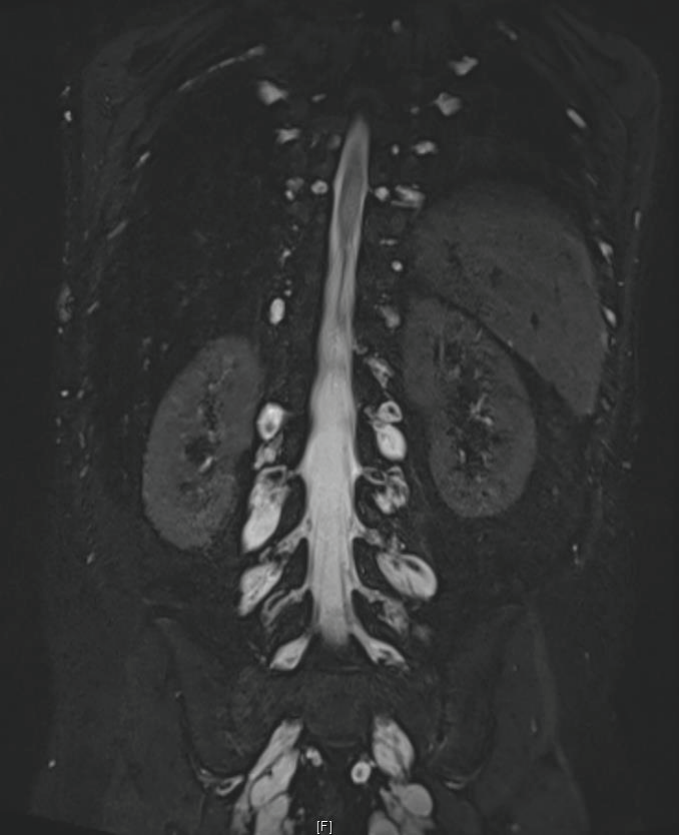
Lumbar neurofibromas: MRI sequence, coronal T2 STIR Gadolinium.

After 10 years of evolution, he presented a gradual degradation of walking with scoliosis. Recently, his condition worsened: walking became impossible and invalidating pain appeared. He was then referred to our department. The neurological examination revealed limb motor deficiency and muscular weakness on C4, predominant on the right side. The patient also reported a net increase in the size of the neurofibroma of the left hand, associated with pain. MRI showed several foraminal staged neurofibromas, along the cervical, thoracic, abdominal and sacral nerve roots (Figure 2). The patient was considered inoperable. We did not perform biopsy analysis and molecular biology analysis of tumours, but given the involvement of the RAS/MEK/ERK pathway in the genesis of neurofibromas, we proposed to the child and his parents to initiate treatment with an MEK inhibitor. Following informed consent, trametinib was started at the dose of 1 mg/day, which had been recommended following the phase I trial [4].

After 1 week of treatment, we found dramatic clinical improvement with walking recovery, and disappearance of pain. A minor decrease in the size of the neurofibroma of the left hand associated with softening of the mass and increased functionality of the hand were reported by the patient. There was no significant radiological improvement on successive MRIs on either the cervical neurofibroma or the optic glioma.

The treatment was well tolerated, apart from cutaneous manifestations, such as transient acnea, bilateral perionyxis in the hands and global mild xerosis of the skin that required short transient interruptions of treatment. Our patient was autonomously able to take his treatment and could resume normal life, including attending school.

The treatment has been stopped after a year. Within 12 months of treatment, we found that improvements had stabilised, and clinical benefits are still ongoing 2 months after stopping the treatment.

## Discussion

We report the case of a young boy with NF1-associated nonmalignant tumours: an optic pathway glioma and multiple neurofibromas. A spectacular clinical improvement after 1 week of treatment, with results maintained over a period of 11 months, was achieved. MRIs performed to evaluate the neurofibromas did not show any reduction in volume following treatment with an MEK inhibitor. However, since there was no decrease in the size of the cervical neurofibroma, and given the fact that the hand neurofibroma had softened, we hypothesised that the improvement of the symptoms was related to a decrease in the pressure made by the softened neurofibroma on neurological structures.

Although cutaneous and subcutaneous neurofibromas and plexiform neurofibromas are benign tumours associated with NF1, the RAS/RAF pathway is involved in their genesis [5]. In a phase 1 trial involving children with inoperable plexiform neurofibroma, selumetinib had acceptable rates of dose-limiting toxic effects when administered on a long-term basis and was associated with a sustained reduction in tumour volumes in the majority of the patients [6]. Interestingly, in the patient we report here, the optico-chiasmatic glioma, which was asymptomatic and was not progressing, did not respond to treatment, while 40% of low-grade gliomas in NF1 patients respond to MEK inhibition [9]. Secondary toxic effects in patients treated with MEK inhibitors appeared mainly cutaneous [10] as was the case in the patient we report here.

Therapeutic options for patients with neurofibromas or plexiform neurofibromas that cannot be surgically removed are limited [11]. Indeed, although many agents have been tested or are being tested, such as mTOR inhibitors and anti-angiogenic agents [11], only imatinib has been shown to reduce plexiform neurofibroma growth by ≥20% in 17% of treated NF1 individuals [12].

An important issue is the duration of treatment. The optimal duration of treatment with MEK inhibitors, and the question of whether or not neurofibromas can resume their growth and can become symptomatic again after stopping treatment, both remain unanswered questions. We have arbitrarily decided to stop treatment after 1 year since the maximum clinical benefit seemed to have been achieved, and we have not observed regrowth after a very short follow-up. Slow tumour regrowth after reaching the maximum response has been observed in several patients, who had required at least one dose reduction as a result of toxic effects [6]. The optimal duration of treatment, as well as long-term follow-up, is crucial issues that must be further investigated.

We do not believe that MEK inhibitor treatment will provide a substitute for standard antalgic treatment for patients with NF1, given the potential toxicity, the cost and the potential duration of treatment. When other symptoms, such as those seen in the patient reported here, are present and cannot be improved by other treatments, then the use of MEK inhibitors may represent a valuable alternative.

## Conclusion

Our case illustrates the potential of MEK inhibitors beyond plexiform neurofibroma and low-grade glioma in patients with NF1, in whom they can lead to rapid control of the symptoms and disease stabilisation.

## Conflicts of interest

The authors have no conflict of interest to declare.

## Funding

This work is not supported by any funding.
